# Editorial: Immunity in aging and age-related diseases and dysfunctions

**DOI:** 10.3389/fimmu.2025.1673414

**Published:** 2025-08-08

**Authors:** Xin Zhang, Calogero Caruso

**Affiliations:** ^1^ Duke Molecular Physiology Institute, Duke University School of Medicine, Duke University, Durham, NC, United States; ^2^ Department of Orthopaedic Surgery, Duke University School of Medicine, Duke University, Durham, NC, United States; ^3^ Laboratory of Immunopathology and Immunosenescence, Department of Biomedicine, Neurosciences and Advanced Diagnostics, University of Palermo, Palermo, Italy

**Keywords:** extracellular vesicles, aging, immunity, mitochondria, cytokines

Aging of the immune system, referred to as immunosenescence, is characterized by age-related alterations in both the adaptive and innate immune responses (1, 2). These age-associated immune changes drive the accumulation of senescent cells and the development of the senescence-associated secretory phenotype, characterized by the release of pro-inflammatory cytokines, chemokines, and tissue-damaging enzymes (2). In parallel, aging disrupts the composition and function of extracellular vesicles (EVs), key mediators of intercellular communication that increasingly reflect and propagate systemic inflammatory signals (3). Collectively, these mechanisms contribute to inflammaging, a chronic, sterile, low-grade inflammatory state (1–4). Mounting evidence underscores the immune system pivotal role not only in driving the biological processes of aging but also in shaping the onset, severity, and progression of multiple age-related diseases (5). Immune dysregulation in older adults contributes to increased vulnerability to infections, reduced vaccine responsiveness, and a heightened incidence of chronic degenerative conditions such as atherosclerosis and osteoarthritis (OA), as well as immune-mediated diseases including rheumatoid arthritis (RA) and various forms of cancer (2, 6–8). This Research Topic brings together original research and critical reviews that shed light on the complex interplay between immunity, aging, and age-related diseases as well as how the resulting disorders might be targeted to promote healthy aging.

Among the key hallmarks of aging (9), mitochondrial dysfunction emerges as a critical driver of cellular senescence and chronic inflammation. In this context, Vendrov et al. explored how NADPH oxidase 4 (NOX4) contributes to mitochondrial dysfunction and oxidative stress in macrophages, promoting aging-associated atherosclerosis. Using Apoe⁻/⁻ mice fed a Western diet to model human-like disease, they found that elevated NOX4 increased mitochondrial oxidative stress, driving monocyte recruitment and plaque inflammation. In contrast, NOX4 deficiency in aged Apoe⁻/⁻ mice reduced oxidative stress and promoted pro-resolving macrophage phenotypes. These findings point to NOX4 as a potential therapeutic target to improve mitochondrial function and attenuate atherosclerosis in aging.

Although both aging and immune dysfunction are implicated in both OA and RA, aging is more commonly recognized as a primary risk factor for OA, while immune dysregulation is central to RA pathogenesis. Interestingly, RA is associated with accelerated immune aging (7). Basu et al. reviewed age-related alterations in monocytes and macrophages, comparing healthy aging to premature immune aging observed in RA. Aging increases peripheral blood monocytes, and this expansion is amplified in RA, particularly in the intermediate monocyte subset that is dominant in RA synovial fluid. Age-related changes in cytokine production, phagocytosis, and metabolism of monocytes and macrophages are also implicated in RA progression. However, contradictory findings in the literature highlight the need for further investigation into age-mediated myeloid cell dysfunction in RA.

In addition, Li et al. reviewed the application of biologic therapies in late-onset rheumatoid arthritis (EORA), a distinct RA subtype with unique clinical and pathological features compared to young-onset RA. Their review summarized the targets, mechanisms of action, and clinical outcomes of biologic agents, including cytokine inhibitors (targeting TNF-α, IL-6, IL-17, IL-23), B-cell–targeting therapies (e.g., anti-CD20), co-stimulation blockers (targeting CD28, CD80, CD86), and JAK-STAT inhibitors. Several biologics, such as secukinumab (IL-17 inhibitor), tocilizumab (IL-6 inhibitor), and abatacept (T-cell modulator), demonstrate particular efficacy in EORA. Interestingly, among TNF-α inhibitors, etanercept shows higher drug retention in EORA compared to adalimumab. Despite promising efficacy, challenges remain in translating these therapies into standard care for the older patients.

Beyond RA, OA represents a highly prevalent degenerative joint disease in older people, driven by the interplay of mechanical stress, metabolic imbalance, and inflammation. In this context, Li et al. (in another review) explored the potential of small interfering RNA (siRNA) as a novel therapeutic avenue. By targeting specific genes involved in inflammation, cartilage degradation, cell senescence, and bone remodeling, siRNA offers a precision tool to modulate key pathways in OA. Recent innovations in siRNA delivery systems enhance its translational feasibility, potentially paving the way for gene-silencing approaches in managing OA.

Contributing to the broader understanding of molecular aging, Zhang et al. characterized EVs as age-sensitive biomarkers carrying cytokines and signaling molecules. Their analysis of plasma EVs from healthy adults revealed age-related shifts in cytokine profiles, including decreased IFN-γ in EV-enriched fraction and IL-17A/F and IL-21 in EV-depleted fraction of plasma. Notably, distinct patterns emerged between endo-EV (associated within or on EVs) and exo-EV (remaining in EV-depleted supernatants) compartments, suggesting differential roles in immune modulation. A decline in fibrinogen-positive EVs with age in healthy adults was also observed. Moreover, EVs from young plasma enhanced fibroblast proliferation, implicating EV phenotypes as regulators of tissue homeostasis and potential tools in anti-aging interventions.

Finally, the clinical implications of immune aging were further explored by Zhuang et al., who addressed the well-known disproportionate impact of COVID-19 on older adults (10). They developed a novel prognostic model based on IgG subclass profiles, capable of identifying older patients at heightened risk for severe disease. By correlating immunoglobulin patterns with clinical outcomes, their algorithm supports personalized care and timely interventions, particularly valuable during future infectious outbreaks in geriatric populations.

Collectively, the contributions in this Research Topic underscore the profound impact of aging on immune function, inflammation, and disease susceptibility. Mitochondrial dysfunction, immune cell reprogramming, and altered molecular signaling converge to drive age-related pathologies such as atherosclerosis, RA, and OA. Emerging tools, from biologics and siRNA therapies to EV profiling and IgG-based predictive models, offer promising, personalized strategies for diagnosing and treating chronic and infectious diseases in older people. A deeper understanding of immune aging is essential to improve healthspan and resilience in older populations.
